# In Vitro MCF-7 Cells Apoptosis Analysis of Carboplatin Loaded Silk Fibroin Particles

**DOI:** 10.3390/molecules25051110

**Published:** 2020-03-02

**Authors:** Nanyak Galam, Pinar Tulay, Terin Adali

**Affiliations:** 1 Tissue Engineering and Biomaterials Research Center, Near East University, P.O. Box 99138 North Cyprus, 10 99138 Mersin, Turkey; galamnanyak@gmail.com; 2 Department of Biomedical Engineering, Faculty of Engineering, Near East University, P.O. Box 99138 North Cyprus, 10 99138 Mersin, Turkey; 3 DESAM Institute, Near East University, P.O. Box 99138 North Cyprus, 10 99138 Mersin, Turkey; Pintulay@gmail.com; 4 Department of Medical Genetics, Faculty of Medicine, Near East University, P.O. Box 99138 North Cyprus, 10 99138 Mersin, Turkey

**Keywords:** breast cancer, silk fibroin, carboplatin, MCF-7 cells, ionic gelation method

## Abstract

Breast cancer ranks as the fifth leading cause of death worldwide. Chemotherapy is commonly used directly or as neo-adjuvant therapy for the management of breast cancer with its attendant adverse effects, underscoring the need to develop biocompatible bioactive compounds for pharmacological applications. The aim of this study is to encapsulate carboplatin (CP) with silk fibroin protein (SF) by using an ionic gelation method as a drug carrier system and assess the apoptotic effect on MCF-7 breast cancer cells during in vitro studies. The characterization of silk fibroin encapsulated carboplatin (SFCP) microparticles was analyzed by FTIR spectrophotometer, SEM, Mastersizer, and biodegradation methods. The encapsulation efficiency and release profile of SFCP microparticles were analyzed by an indirect UV–Vis spectrophotometric method. An apoptotic screening of MCF-7 cells was carried out with 10–200 µg/mL CP loaded SFCP, which were cultured for 24, 48, and 72 h. Data were analyzed using the Student’s *t* test and analysis of variance. FTIR and drug release studies confirmed an interaction of silk fibroin with the carboplatin moiety. SFCP showed successful encapsulation of the carboplatin moiety. Apoptotic screening showed a dose dependent increase in absorbance, indicating significant cell death (*p* < 0.05). Thus, the direct apoptotic effect of SFCP microparticles on MCF-7 was confirmed.

## 1. Introduction

Breast cancer is the second most diagnosed cancer in women in both developed and developing countries. It is estimated that over 2.09 million cases exist worldwide and that 627,000 women passed away in 2018 [1]. The most frequently used treatment modalities for breast cancer are surgery and chemotherapy [2,3]. However, there are problems associated with chemotherapeutic therapies as a result of acquired resistance as well as drug toxicity [4]. Thus, designing chemotherapeutic drug delivery systems is very important for effective cancer treatment. In the last decade, studies focusing on targetable drug delivery systems for breast cancer treatment have gained in importance [5,6]. Although the outcomes after breast cancer treatment have improved, the drawbacks such as side effects are still a major issue [7,8]. The pro-drug approach causes a loss of stability and drug activity after modification. Colloidal delivery systems can be used to encapsulate active drugs to improve solubility, stability, and adsorption characteristics of proteins, such as silk fibroin [9]. Recently, colloidal drug carrier systems have become an emerging field for cancer drug delivery systems [7]. Nano- and microparticles act as a tool and enhance intracellular uptake and distribution inside the cancer cell [10,11]. They are believed to take advantage of the heterogeneous nature of the tumor vasculature, which is usually tortuous with widened endothelial gaps and junctions in most regions of the tumor, thus utilizing the enhanced permeation and retention effect (EPR) to ensure a localization of the drugs at the tumor site [12]. The molecules, as well as drugs including nanoparticles, microparticles, and macro drugs, accumulate in the tumor tissue in bigger quantities compared to the normal tissues. This may be due to the leakage of the tumor vasculature contrary to the normal tissue. This EPR effect plays a vital role in the ability of the particles gaining access to the tumor site and remaining there due to the poor lymphatic drainage of the tumor site [13]. A number of anticancer drugs are being used in light of this effect, including doxorubicin loaded liposomes and paclitaxel bound albumin. On the other hand, a number of studies which have been carried out showed that the drug delivery was not significant [13,14]. This implies that particles must be biocompatible and biodegradable in order to avoid an accumulation of material at the tumor site. Silk fibroin (SF) protein has the advantage of being a naturally occurring polymer, which is derived from the *Bombyx mori* (*B. mori*) cocoons of the silk worm. This vital protein is an important biological macromolecule used in the design of anticancer drug delivery systems [15,16]. Due to its biocompatibility [16,17], biodegradability, blood compatibility [18], and modifiability [19], silk fibroin is used for the encapsulation of drug molecules. Polymeric nano- and microparticles can be prepared from natural protein silk fibroin [20]. Silk fibroin particles have not only been used to deliver drugs, but also to deliver genes in the treatment of a number of cancer types [20]. More particularly, silk fibroin particles have not been used to deliver carboplatin, the subtype of cisplastin, into the breast cancer cells. 

**Figure 1:**
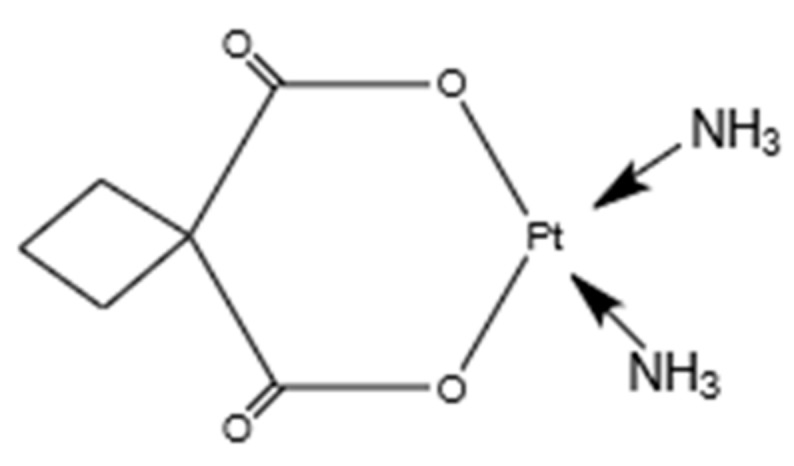
Molecular structure of carboplatin (C_6_H_12_N_2_O_4_Pt).

Carboplatin (CP), a platinum anticancer drug, is used to treat many types of human cancer. A structural feature of carboplatin, as shown in Figure 1, comprises a bidentate dicarboxylate chelate leaving ligand, which makes it much less chemically reactive than the first-generation platinum anticancer drug cisplatin [21,22]. Carboplatin exerts its biological effects by interacting with genomic DNA and proteins. 

To date, a number of methods have been applied to prepare nano- and microparticles. The electrospray method is an example of these methods [23]. However, it has the drawback of requiring a lot of solvents and surfactants, thus making the process very expensive. Furthermore, it leads to generating a lot of residue, which may be toxic to the environment with minimal yield of the required drug [24]. The aim of this study was to encapsulate carboplatin with silk fibroin protein by using the ionic gelation method and evaluate a targeted apoptotic effect on breast cancer cell line MCF-7. The characterization of silk fibroin encapsulated carboplatin (SFCP) microparticles was analyzed by FTIR, scanning electron microscopy, Mastersizer, and biodegradation methods. The loading capacity and release profile of SFCP microparticles were analyzed by an indirect method by using a UV–Vis spectrophotometric method at 450 nm absorption wavelength. The apoptotic effect of 10–200 µg/mL CP loaded SFCP microparticles on MCF-7 cells was investigated following 24, 48, and 72 h of culturing using the Apostrand Elisa kit. 

## 2. Results and Discussions

### 2.1. The Synthesis and Characterization of Silk Fibroin Encapsulated Carboplatin Particles

The main aim of this study was to develop silk fibroin carboplatin microparticles as a platform for targeted drug delivery using the ionic gelation method. Although other methods have been used to encapsulate carboplatin, there were no readily available data in regards to encapsulating the drug using the ionic gelation method. The SFCP microparticle size distribution analysis was carried out by Mastersizer within the size range of 0.02–2000 µm with normal sensitivity and 12.24% obscuration. The concentration of the SFCP microparticles was 0.0728% volume. Volume weighted and surface weighted mean values are listed on Table 1 as 40.629 µm and 79.695 µm, respectively.

**Figure 2:**
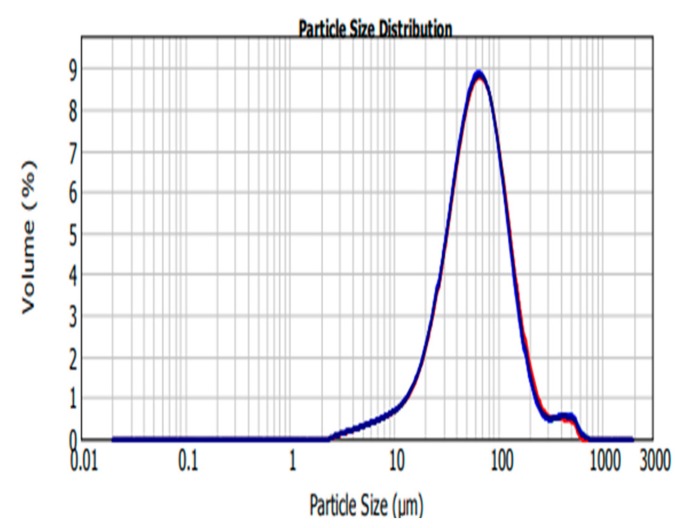
Silk fibroin encapsulated carboplatin (SFCP) microparticles size distribution graph, volume (%) versus particle size (µm) analyzed by MasterSizer 2000.

The size ranges of SFCP microparticles are larger than the size obtained by Subia and colleagues [25] and falls within the microparticle range as discussed by Singh and colleagues [26]. A study performed by Senchova and colleagues analyzed the vascular diameter and morphology of breast cancer tumors and suggested the average peritumoral and intratumoral capillary diameter as being between 87.8 microns and 64.4 microns with certain areas lacking endothelial linings [27], thus particles of this size are said to have the capacity to extravagate into the tumor, and utilize an EPR effect to ensure targeted drug delivery in the tumor cells [28]. In this study, 50% of SFCP microparticles are below 61.728 µm and 90% are below 146.080 µm as shown in Table 1 and Figure 2. This supports a utilization of an EPR effect of SFCP microparticles to ensure targeted drug delivery in the tumor cells.

**Figure 3:**
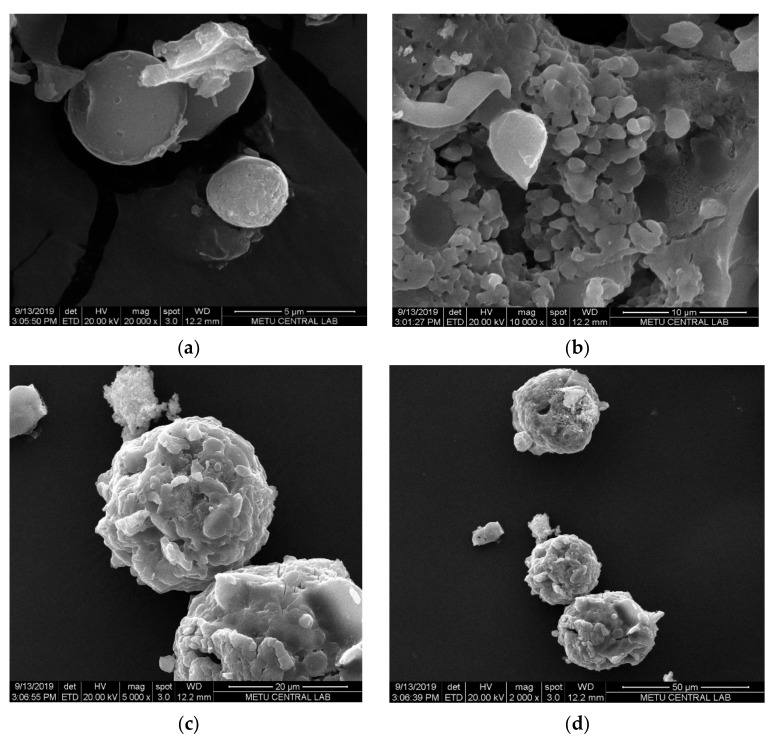
SEM micrographs of SFCP microparticles at (**a**) 5 µm, (**b**) 10 µm (**c**), 20 µm, and (**d**) 50 µm scales.

Morphological analysis was carried out by SEM. The SEM micrographs of SFCP microparticles showed the particles were spherical in shape (Figure 3a), but they were not uniform with a size range of 10–146.08 µm. This result was supported by the Mastersizer analyses of SFCP microparticles. The globules, as shown in Figure 3b, are made up of small pseudospherical particles that form aggregates (Figure 3c,d).

### 2.2. Biodegradation Analysis

The SFCP particles were tested against the protease solution (0.2 mg/100 mL H_2_O) at 37 °C. The experiment was repeated three times. The results are shown in Table 2. The particles were synthesized, with a varying amount of carboplatin, whilst keeping the amount of silk fibroin constant (Table 3). It is clear that the gelation decreases the biodegradation of the SFCP microparticles. As the amount of silk fibroin increases with respect to carboplatin, it enhances the crosslinking and affects the rate of biodegradation. These results indicate that the biodegradation rate may be controlled by either varying the amount of silk fibroin or carboplatin in the reaction.

### 2.3. FTIR Spectra Analysis

**Figure 4:**
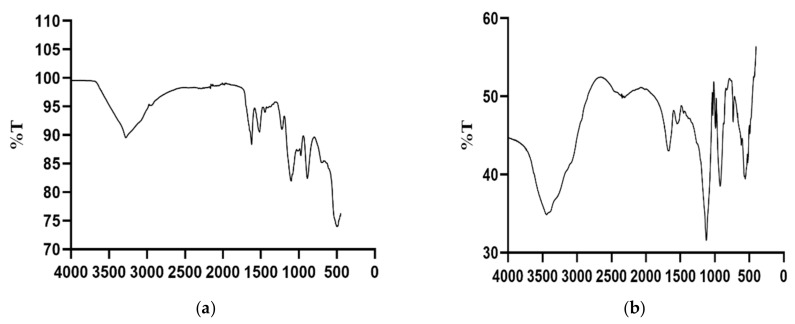
(**a**) FTIR spectrum of silk fibroin protein (SF) microparticles; (**b**) FTIR spectrum of SFCP microparticles. X-axis corresponding to λ (cm^−1^).

FTIR spectra showed a characteristic silk fibroin signature. The FTIR spectra results are consistent with that of silk fibroin and carboplatin and are in agreement with previous studies [29,30]. The increased absorbance shown by the SFCP particles may be due to the presence of a ring like structure of carboplatin, thus increasing its molecular absorptivity. The tracing of SFCP particles generally showed more absorptivity and less transmittance compared to that of silk fibroin particles alone. They both showed peaks suggestive of an OH group at 3452 cm^−1^ for SFCP microparticles as shown in Figure 4a and 3274 cm^−1^ for silk fibroin microparticles alone as shown in Figure 4b. A peak denoting a carbonyl functional group is present at 1680 cm^−1^ for SFCP microparticles and 1622 cm^−1^ for SF particles suggestive of the presence of amide 1. A secondary NH bend is observed at 1554 cm^−1^ in the SFCP particles and around 1517 cm^−1^ for the plain SF particles. The carbonyl peak is present at around 1123 cm^−1^ for the SFCP microparticles. These spectral features and shifts are characteristic of carboplatin silk fibroin interactions [29].

### 2.4. Percentage Encapsulation of SFCP Microparticles

**Figure 5:**
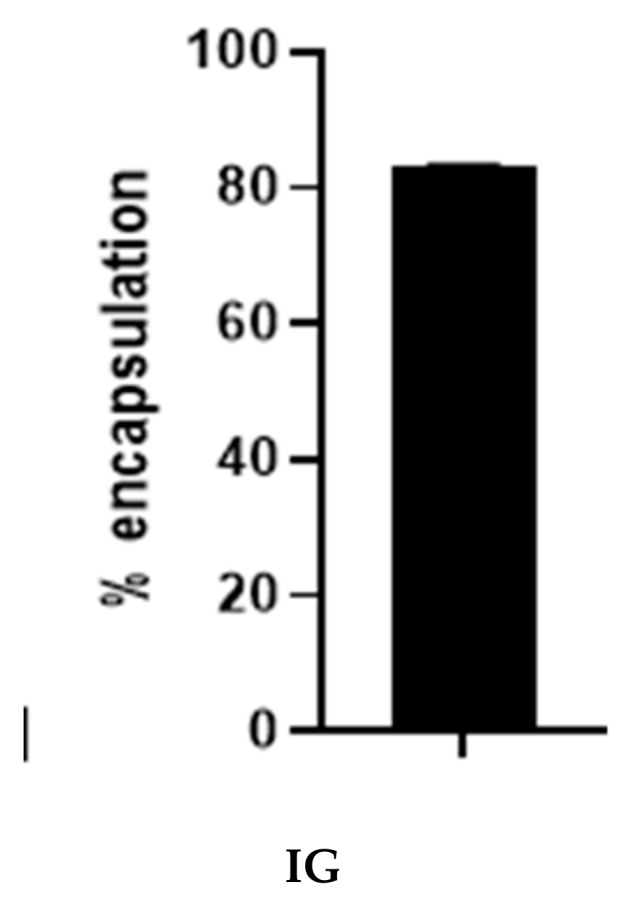
Percentage encapsulation of SFCP microparticles using the ionic gelation method (IG).

The percentage encapsulation was determined by measuring the weight of the drug in the particle and dividing it with the feeding drug via an indirect method described by Basotra and colleagues [31]. Figure 5 shows the mean percentage encapsulation, including the standard deviation of particles synthesized via the ionic gelation method. The mean encapsulation of the ionic gelation method was 83.32 ± 0.07%. The result is highly impressive in comparison to previous encapsulation studies using carboplatin [16]. The ionic gelation method of encapsulating carboplatin into silk fibroin protein may reduce drug waste and increase the concentration of the drug in the particle.

### 2.5. Cummulative Drug Release

**Figure 6:**
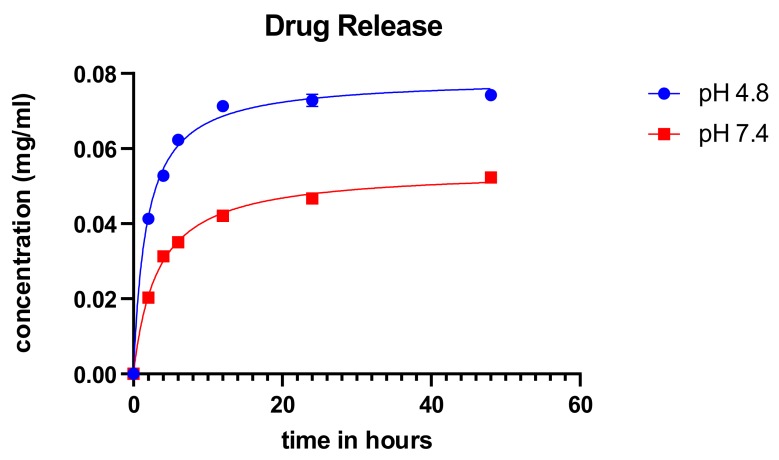
Cumulative drug release of carboplatin from SFCP microparticles. X-axis corresponds to time (in hours).

The cumulative drug release was measured for over 48 h at different pH for the synthesized particles using the ionic gelation method as shown in Figure 6. The particles showed an initial burst release of the drug and subsequently stabilized over time. There was a release of a higher concentration of the drug in acidic pH. The characteristic burst release with subsequent normalization of the concentration from particles is consistent with the findings of Perez and colleagues [30]. These results corroborate with the study performed by Subia et al. [25] and suites the profile for candidate anticancer treatment modality since the cancer microenvironment is said to be acidic.

### 2.6. Apoptotic Activity of SFCP Particles

**Figure 7:**
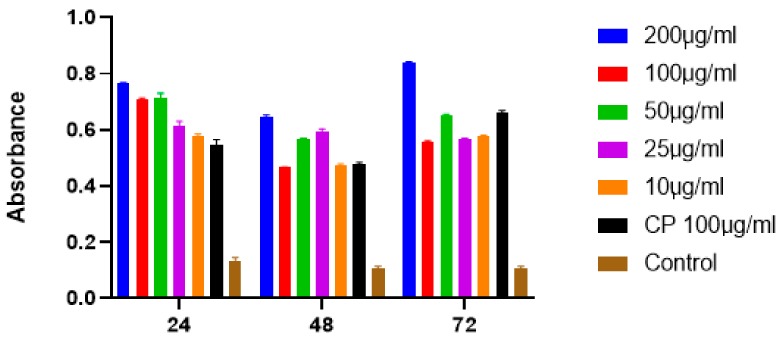
The graphs showing the results of one-way ANOVA analysis of SFCP on MCF-7 cell lines at 24, 48, and 72 h.

The absorbance data were analyzed by using the Student’s *t* test and one-way ANOVA. These results show the incubation of SFCP on MCF-7 cell lines at time intervals of 24, 48, and 72 h at 450 nm absorbance. SFCP particles prepared by the ionic gelation method showed significant apoptotic activity at a 95% confidence interval when compared with the control (Figure 7). There were no significant differences amongst different incubation times. This may indicate a sustained activity of the drug over the 72-h period. The IC_50_ of SFCP particles was 9.23 µg/mL (graphpad prism version 8).

The absorbance (at 450 nm) is directly proportional to the apoptotic activity, which was shown to be dose dependent with an optimal activity at 72 h. All concentrations of SFCP microparticles showed statistical significance compared to the control (*p* < 0.0001). However, there was no significant difference in the apoptotic activity between the different concentrations of SFCP microparticles. Since apoptosis has been shown to be involved in the development of cancers [32], any process that could induce apoptosis in cancers may as well reverse the proliferation of cancer cells. Silk fibroin particles have been used in several studies as carriers for anticancer drugs. Moreover, silk fibroin has also been used to encapsulate natural products as well as anticancer drugs to prevent the proliferation and to induce apoptosis in breast cancer cells [33,34]. This is the first study to encapsulate the anticancer drug carboplatin with SFCP using the ionic gelation method that showed significant apoptotic activity at a 95% confidence interval when compared with control. Furthermore, the results indicate a sustained activity over a three-day period. This confirms that the SFCP microparticles are an effective apoptotic agent against MCF-7 breast cancer cells.

## 3. Material and Methods

### 3.1. Materials

The cocoons of the silk fibroin from the *Bombyx mori* silk worms were obtained from North Cyprus. The Apostrand Elisa apoptosis kit (Enzo, Switzerland), MCF-7 breast cancer cells (ATCC® HTB-22™, Manassas, Virginia, US), 10% fetal bovine serum (FBS Thermofisher, Grand island, Newyork, US), 1% penicillin/streptomycin (Thermofisher, Grand island, US), insulin in DMEM/F12 (Thermofisher US), carboplatin (C_6_H_12_N_2_O_4_Pt, Sigma Aldrich, Schnelldorf, Germany), sodium carbonate (Na_2_CO_3_, Sigma Aldrich, Germany), calcium chloride (CaCl_2_, Sigma Aldrich, Germany), ethanol (C_2_H_5_OH, Sigma Aldrich, Germany), and dialysis membrane (cut off MW 12,400) with a full average diameter of 16 mm, a flat width of 25 mm, and a capacity of 60 mL/ft were purchased from Sigma Aldrich, Germany. All other reagents were of analytical grade and also purchased from Sigma Aldrich, Germany.

### 3.2. Methods

#### 3.2.1. Purification of Silk Fibroin

The silk cocoons were cut into small pieces and degummed with a 0.1 M Na_2_CO_3_ solution at 70 °C for three sessions at a speed of 1.5 rpm. After each degumming session, SF was washed with ultra-pure water and dried at room temperature. The dissolution was then carried out by using a strong electrolyte solution (molar ratio of n_H2O_:n_CaCl2_:n_CH3CH20H_; 8:2:1) at 70 °C. Dialysis of the solution was performed by using a snake skin dialysis membrane against ultra-pure water for further purification of the SF protein.

#### 3.2.2. Synthesis of SFCP Particles and Encapsulation of Carboplatin

Two methods were utilized for the synthesis of silk fibroin encapsulated carboplatin microparticles to be able to find the optimum encapsulation and drug release efficiency of SFCP microparticles. In the first method, the desired amount of the carboplatin drug (10 mg/mL) was blended with 10 mL of pure silk fibroin protein solution and stirred for 30 min at 1.5 rpm on a magnetic stirrer. Then, the blended solution was added to 0.1 M sodium triphosphate pentabasic (TPP) solution by the dropwise technique, and the SFCP microparticles were prepared by using an ionic gelation method.

The mixture was ultra-sonicated (Sonics Vibro-cell) and centrifuged at 400 rpm for 10 min. The aggregates were collected and filtered through a micrometer-gauged syringe for further use. The supernatant was collected separately and analyzed by an indirect method using spectroscopy to determine the amount of unbound carboplatin as validated by Basotra [31]. Briefly, a known amount of carboplatin was used as the standard. Then, aliquots of the supernatant were collected and conjugated to O-phenylenediamine to enhance the molecular absorptivity of carboplatin in the UV region. The amounts of the unbound drug in the supernatant were measured using a simple spectroscopic method. The value was subtracted from the feeding drug to determine the amount of the drug in the microaggregates. Each sample was analyzed in triplicates [31]. The collected microparticles were washed with ultra-pure water followed by lyophilization by freeze drying at −20 °C (Christ, Alpha 1-4 LD plus) to aid the stability of the particles [32,33]. A dry sterilization process was used at the Near East University Hospital Sterilization Unit for sterilizing SFCP microparticles [34].

Three samples with different proportions were prepared as shown in Table 1. Percent encapsulation was calculated by Equation (1) as follows:

(1) $$\%Encapsulation\;=\;\frac{\left(weight\;of\;drug\;in\;microparticle\right)}{\left(weight\;of\;drug\right)}\;\times \;100.$$

#### 3.2.3. Particle Size Analysis of SFCP Particles

The particle size analysis of SFCP microparticles was carried out by using Matersizer 2000 Ver. 5.60 Serial no: MAL100704 (Malvern Instruments Ltd., Malvern, UK.) from the Middle East Technical University (METU) Central Laboratory located in Ankara, Turkey.

#### 3.2.4. Morphological Analysis of SFCP Microparticles

The particles were vacuum coated with gold microparticles and surface morphology was observed with a scanning electron microscope (S—3400 N: Hitachi, Japan). The photograph was taken at a voltage of 15 KV. All the procedures were performed at room temperature. The sizes of the particles and distribution were determined using a Mastersizer 2000 (Malvern, UK).

#### 3.2.5. Biodegradation Tests for SFCP Particles

A solution of protease (0.02 mg/100mL) was used at 37 °C for analyzing biodegradation behavior of SFCP microparticles. The biodegradation percentage was calculated by using Equation (2):

(2) $$Biodegradation\;\left(weight\;loss\right)\%=\;\frac{weight\;\left(t\right)}{weight\;\left(0\right)\;}\;\times \;100,$$

where:

*weight (t)* = weight after given time;

*weight (0)* = weight of particles before biodegradation.

#### 3.2.6. Fourier Transform Infrared Spectroscopy (FTIR) Analysis

The FTIR spectrum of SFCP microparticles was carried out by using Perkin Elmer Spectrum Two at Eastern Mediterranean University, North Cyprus, to assess the conformation and transition of the silk fibroin microparticles as well as the entrapment of carboplatin.

#### 3.2.7. Drug Release ANALYSIS

The in vitro release profile was assessed via a dialysis method. The release profile of carboplatin from the silk fibroin was dialyzed against phosphate buffer saline (PBS) at room temperature. This was performed by using two different pH, an acidic pH of 4.8 that was comparable with the pH of the cancer microenvironment, and a pH of 7.4 comparable to the physiological pH of normal cells in vivo. The particles were sealed in a dialysis membrane of MCO 10,000 and immersed in 50 mL of external buffer solution. The dialysis was continued and the drug release monitored for over 48 h by an indirect spectroscopic method as described by Basotra et al. [31]. An aliquot (2 mL) of the dialyzed fluid was collected with a replacement at different time intervals and as described in the drug release section. It was conjugated with O-phenylenediamine to yield a green color and increase the molar absorptivity of the released drug to enable its determination via UV spectroscopy using Beer–Lambert’s law [31].

(3) $$\%\;Drug\;Release=\;\frac{Weight\;of\;drug\;in\;nanoparticle}{Weight\;of\;nanoparticles}\;\times \;100$$

#### 3.2.8. Culturing MCF-7 Breast Cancer Cell Lines

The purchased MCF-7 cells (ATCC® HTB-22™, Manassa, Virginia, US) were cultured in 10% FBS, 1% penicillin/streptomycin, and 4 mg insulin in DMEM/F12 in a humidified atmosphere of 5% CO_2_ at 37 °C. The cells were observed and the culture media were changed every other day. Once the cells reached 70% confluence, they were passaged.

#### 3.2.9. Assessment of Apoptosis

Apoptosis was assessed using the Apostrand Elisa apoptosis kit (Enzo, Switzerland). This kit assays the sensitivity of DNA in apoptotic cells to formamide denaturation, and this was performed with the aid of a monoclonal antibody to single strand DNA. Approximately 5000 cells were seeded in each well of a 96 well plate. After 24 h, different concentrations of SFCP microparticles were added to each well. The cells were incubated with 10, 25, 50, 100, and 200 µg/mL SFCP microparticles for 24, 48, and 72 h, respectively. Cells were cultured in carboplatin (100 µg/mL) as the positive control, whilst only culture media were added as the negative control. The absorbance at 450 nm was recorded with the aid of a microplate reader and the IC_50_ was calculated with the aid of the graphpad prism statistical tool version 8.

#### 3.2.10. Statistical Analysis

All the data obtained were expressed as mean ± standard error of mean. Significant differences were tested by a Student’s *t* test with a statistical significance set to a probability level of 0.05. Moreover, Anova test was also used for statistical analysis using the graphpad prism 8.1 and graphpad in stat 3.0 statistical tools.

## 4. Conclusions

Although this study is limited by the use of one breast cancer cell line, it has shown that SFCP particles can be successfully prepared using the ionic gelation method for the first time. Its encapsulation was confirmed via an indirect method described by Basotra [31], and an FTIR also showed the characteristic features of SF interacting with carboplatin. The cumulative drug release profile was similar with the other anticancer drugs favoring an acidic microenvironment, which is important for passive drug targeting. The anti-apoptotic activity showed statistical significance in MCF-7 breast cancer cells compared to the controls. This effectiveness may be related to the slow release profile of SFCP microparticles over 72 h. Therefore, SFCP microparticles made via the ionic gelation method are promising in the targeted drug delivery system for breast cancer. Future studies using different cell lines, such as metastatic types, will provide further information on the effectiveness of SFCP in drug delivery.

## Figures and Tables

**Table 1:** Particle size distribution results of Mastersizer analysis.

SFCP Microparticle Size Distribution Analysis	
Weighted residual	0.802%
Size Range	0.02–2000 µm
Sensitivity	Normal
Concentration	0.0728% Volume
Specific Surface Area	0.184 m^2^/g
Surface weighted mean D [3,2]	40.629 µm
Volume weighted mean D [4,3]	79.695 µm
d(0.1)	22.212 µm
d(0.5)	61.728 µm
d(0.9)	146.080 µm

**Table 2:** The biodegradation test of SFCP microparticles in protease solution at 37 °C.

Time (h)	SPCP1 (µg)	SPCP2 (µg)	SPCP3 (µg)
**0.00**	100.00	100	100
**0.25**	90.88	86.30	83.40
**0.50**	82.64	79.22	75.76
**0.75**	73.40	69.85	66.41
**1.00**	60.87	56.20	50.22
**1.25**	59.41	49.23	42.35
**1.50**	52.73	43.32	30.72
**1.75**	45.53	38.90	13.65
**2.00**	36.90	30.24	05.44
**2.30**	27.23	18.84	0
**2.75**	18.35	05.67	0
**3.00**	04.23	0	0
**12**	0	0	0
**24**	0	0	0
**25**	0	0	0
**26**	0	0	0

**Table 3:** Fibroin–carboplatin microparticles synthesis conditions for the ionic gelation method.

Sample	SF (mL)	CP (mL) (10%) *v*/*v*)	0.1 M TPP (mL)
SFCP1	10	0.01	50
SFCP2	10	0.05	50
SFCP3	10	0.10	50

SF: silk fibroin; CP: carboplatin; TPP: sodium triphosphate pentabasic.
